# Evaluating initial responses to brolucizumab in patients undergoing conventional anti-VEGF therapy for diabetic macular edema: a retrospective, single-center, observational study

**DOI:** 10.1038/s41598-023-37726-5

**Published:** 2023-07-05

**Authors:** Takao Hirano, Akane Kumazaki, Ryuji Tomihara, Syun Ito, Ken Hoshiyama, Toshinori Murata

**Affiliations:** 1 Department of Ophthalmology, Shinshu University School of Medicine, 3-1-1 Asahi, Matsumoto, Nagano 390-8621 Japan; 2 Department of Ophthalmology, Suwa Red Cross Hospital, Suwa, Nagano Japan

**Keywords:** Retinal diseases, Diabetes complications

## Abstract

Our retrospective, single-center, observational study aimed to evaluate the initial responses to intravitreal injection of brolucizumab (IVBr) in patients undergoing anti-vascular endothelial growth factor (VEGF) therapy for diabetic macular edema (DME). In total, 23 eyes of 20 patients with DME treated with at least one intravitreal injection of ranibizumab or aflibercept within one year and then switched to IVBr were included. Best corrected visual acuity (BCVA), central macular thickness (CMT), and macular volume (MV) on optical coherence tomography images were evaluated just before the most recent conventional anti-VEGF (ranibizumab/aflibercept) injection therapy (V1), one month after the most recent traditional anti-VEGF therapy (V2), just before the first IVBr (V3), and one month after the first IVBr (V4). BCVA, CMT, MV, and presence of intraocular inflammation (IOI) were evaluated at each visit. Anterior chamber flare values were also examined at V3 and V4. BCVA showed significant improvement at V2 (0.30 ± 0.23) than V1 (0.39 ± 0.29) and at V4 (0.34 ± 0.26) than V3 (0.48 ± 0.34) (*P* = 0.002, *P* < 0.001). However, no significant difference was observed between V2 and V4 (*P* = 0.257). CMT was significantly thinner at V2 (346.8 ± 90.2 µm) than V1 (495.5 ± 123.8 µm), and at V4 (322.2 ± 95.7 µm) than V3 (536.5 ± 166.0 µm) (*P* < 0.001, *P* < 0.001), but no significant difference was observed between V2 and V4 (*P* = 0.140). MV was significantly smaller at V2 (11.6 ± 2.0 mm^3^) than V1 (12.6 ± 1.9 mm^3^) and at V4 (11.2 ± 2.0 mm^3^) than V3 (12.6 ± 2.0 mm^3^) (*P* < 0.001, *P* < 0.001), and even significantly smaller at V4 than V2 (*P* = 0.009). No patient had IOI. No significant changes were observed in anterior chamber flare values between V3 and V4 (25.6 ± 14.6 vs. 24.0 ± 11.5 photon count/ms; *P* = 0.543). Both CMT and MV significantly reduced without any adverse events one month after switching from conventional anti-VEGF to IVBr therapy for DME, including IOI. MV was significantly lower for IVBr than anti-VEGF therapy after one month of treatment. Therefore, brolucizumab may be a viable treatment option for DME patients considering switching from conventional anti-VEGF agents for various reasons, such as poor response or inability to extend dosing intervals.

## Introduction

Diabetic macular edema (DME) is a leading cause of vision loss in the working population^1^. Landmark randomized clinical trials (RCTs) of ranibizumab^2^ and aflibercept^3^ reported that anti-vascular endothelial growth factor (VEGF) therapy was more effective in the management of DME than previous mainstream focal/grid laser or topical steroid therapies. Anti-VEGF therapy has since become the first-line treatment for DME^4,5^. However, the aforementioned extended RCTs also revealed that several patients were refractory to anti-VEGF therapy or required frequent drug administration^6,7^. To overcome these limitations, a combination therapy of anti-VEGF treatment and focal/grid photocoagulation was introduced. Promising results have been reported with the use of this combination therapy^8,9^. Significant anatomical improvement was reported by a study after switching to aflibercept for persistent DME refractory to bevacizumab or ranibizumab^10^. Brolucizumab as a single-chain antibody fragment was approved for the treatment of DME in June 2022 in Japan. Two clinical trials for DME, KESTREL and KITE, showed non-inferiority of brolucizumab to aflibercept in terms of visual outcomes, with more study participants achieving central macular thickness (CMT) < 280 µm^11^. Although favorable real-world results of switching to brolucizumab have been reported for age-related macular degeneration in patients previously treated with anti-VEGF therapy^12,13^, those for DME are not available. In addition, safety signals for brolucizumab in age-related macular degeneration (AMD) have been reported in RCTs and post hoc analyses, including intraocular inflammation (IOI) and retinal vasculitis with or without vessel occlusion^14–17^. Although the results of RCTs suggest that IOI associated with brolucizumab is not extremely common in patients with DME compared to AMD^11,14^, a case of IOI with vascular occlusion after intravitreal injection of brolucizumab (IVBr) for DME has been reported^18^. This study evaluated the short-term response and safety of IVBr in patients switching from conventional anti-VEGF therapy for DME.

## Methods

### Ethical considerations

This study was approved by the Ethics Committee of Shinshu University Hospital (ID number: 5820) and adhered to the tenets of the Declaration of Helsinki.

Written informed consent was not obtained because the study was retrospective. The need for informed consent is waived by the Ethics Committee of Shinshu University. The university website provided participants with an opportunity to opt out of the study.

### Study design and procedure

This was a retrospective, single-center, observational study. DME patients treated with at least one intravitreal injection of aflibercept or ranibizumab in the prior year who were switched to IVBr between July 1, 2022, and February 28, 2023, and followed for at least one month were enrolled in this study.

**Figure 1:**
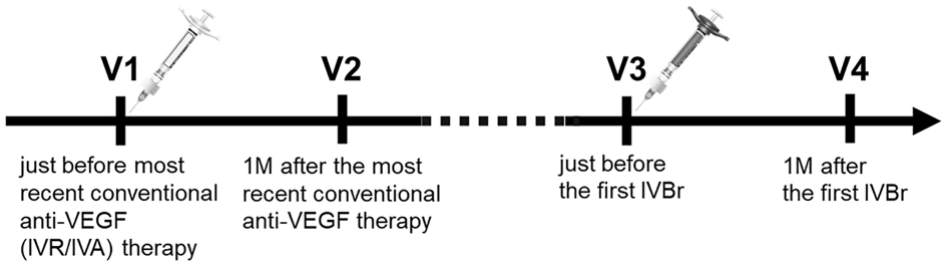
Timepoints evaluated in this study. Patients were evaluated at four-time points: V1: just before the last conventional anti-VEGF (ranibizumab/aflibercept) injection, V2: one month after the previous injection, V3: just before the first IVBr, and V4: one month after the first IVBr. IVBr, intravitreal injection of brolucizumab; VEGF, vascular endothelial growth factor.

We analyzed the medical records of the study participants at four different time points (Fig. 1): just before the most recent conventional anti-VEGF (ranibizumab/aflibercept) therapy (V1), one month after the most recent conventional anti-VEGF therapy (V2), just before the first IVBr (V3), and one month after the first IVBr (V4). Best-corrected visual acuity (BCVA) was determined, and complete ophthalmic examination, including a slit-lamp test and fundoscopy following pupil dilation, was performed at each visit. Signs of IOI and/or retinal vasculitis were recorded if present. Retinal imaging was performed at each visit using an ultra-wide-field fundus imaging system (CLARUS 500™, Carl Zeiss Meditec Inc., Dublin, CA) and spectral-domain optical coherence tomography (SD-OCT, Cirrus HD-OCT, Carl Zeiss Meditec, Inc., Dublin, CA), and macular cube 512 × 128 scans were obtained. The macular cube 512 × 128 scan uses a raster scan mode that scans a 6 × 6-mm macular area into 512 × 128 (length by width) points. CMT (mean retinal thickness of 1-mm diameter circle) and macular volume (MV) (retinal volume of 6 × 6 mm) were evaluated from the acquired OCT images. Laser flare meter was used to measure anterior chamber flare values (FC-700, Kowa Co. Ltd, Tokyo, Japan) at V3 and V4.

### Statistical analysis

BCVA measured using the Landolt chart was converted into logarithm of the minimum angle of resolution (log MAR) values for statistical analyses. Paired t-test was used to determine the significance of the difference between the values before and after treatment. Statistical significance was set at *P* < 0.05. All statistical analyses were conducted using GraphPad Prism version 9.2.1 (GraphPad Inc).

### Meeting presentation

Fuji Retina, Tokyo, Japan, 2023.

## Results

### Baseline characteristics of the patients and their eyes

Table 1 summarizes the baseline characteristics of the patients and their eyes. Twenty-three eyes from 20 patients with DME were included in this study. The mean age of the study population was 67.4 ± 10.7 years, and 16 patients were male (80.0%). The mean hemoglobin A1c (HBA1c) level was 7.0 ± 0.7%, and the mean estimated glomerular filtration rate (eGFR) was 65.9 ± 32.4 mL/min/1.73 m^2^. Of the 23 eyes, four had moderate non-proliferative diabetic retinopathy (NPDR), 13 had severe NPDR, 6 had proliferative diabetic retinopathy (PDR), and 17 (73.9%) had been treated with pan-retinal photocoagulation (PRP).

**Table 1:** Baseline characteristics of the patients with diabetic macular edema treated with anti-vascular endothelial growth factor agents and switched to brolucizumab.

Characteristic	
Per patient n = 20	
Age, mean ± SD, years	67.4 ± 10.7
Sex, n (female/male)	4/16
HBA1c, mean ± SD, %	7.0 ± 0.7
eGFR, mean ± SD, mL/min/1.73m^2^	
Per eye n = 23	
DR severity, n (mild/moderate/severe NPDR/PDR)	0/4/13/6
Previous PRP (with/without), n	17/6
Anti-VEGF agent before switching to IVBr (ranibizumab/aflibercept/ranibizumab and aflibercept), n	3/19/1
Number of anti-VEGF treatments before switching to IVBr, mean ± SD, times	11.6 ± 13.4
Interval between last anti-VEGF treatment before switching and first IVBr, mean ± SD, months	6.0 ± 3.9

DR, Diabetic retinopathy; eGFR, estimated glomerular filtration rate; IVBr, intravitreal injection of brolucizumab; NPDR, non-proliferative diabetic retinopathy; PDR, proliferative diabetic retinopathy; PRP, pan-retinal photocoagulation; SD, standard deviation; VEGF, vascular endothelial growth factor.

Reasons for switching to IVBr were as follows: inadequate efficacy (once the dose did not fall below 315 µm) in 9 eyes; extended dosing interval (once the dosing interval did not exceed three months) in 6 eyes; and others (due to expectation of a new drug/desire to restart treatment) in 8 eyes. Conventional anti-VEGF treatment before switching to IVBr included ranibizumab in 3 eyes, aflibercept in 19 eyes, and both in 1 eye. The mean number of anti-VEGF treatments before switching to IVBr was 11.6 ± 13.4, and the interval between the last anti-VEGF treatment before switching and the first IVBr was 6.0 ± 3.9 months.

### Visual and anatomical outcomes

**Figure 2:**
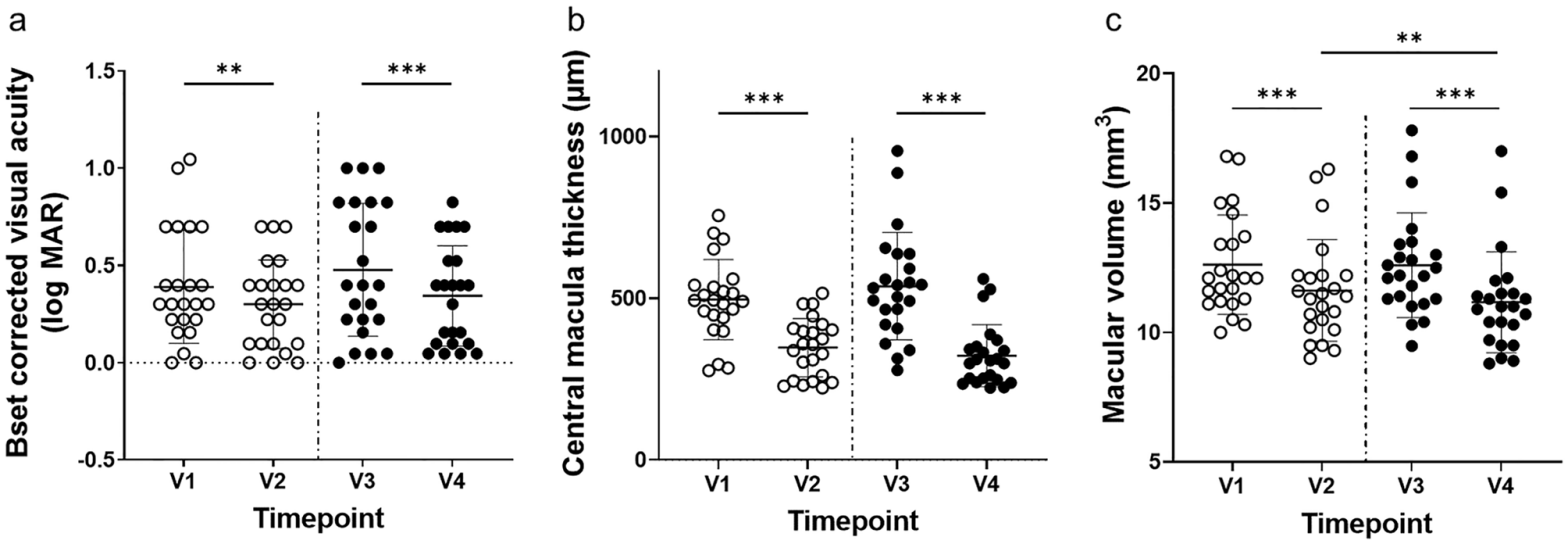
BCVA (log MAR), CMT, and MV at each timepoint. (**a**) BCVA showed significant improvement at V2 (0.30 ± 0.23) than at V1 (0.39 ± 0.29) and at V4 (0.34 ± 0.26) than at V3 (0.48 ± 0.34) (*P* = 0.002, *P* < 0.001), but no significant difference was observed between V2 and V4 (*P* = 0.257). (**b**) CMT was significantly thinner at V2 (346.8 ± 90.2 µm) than at V1 (495.5 ± 123.8 µm) and at V4 (322.2 ± 95.7 µm) than at V3 (536.5 ± 166.0 µm) (*P* < 0.001, *P* < 0.001); however, no significant difference was observed between V2 and V4 (*P* = 0.140). (**c**) MV was significantly smaller at V2 (11.6 ± 2.0 mm^3^) than at V1 (12.6 ± 1.9 mm^3^) and at V4 (11.2 ± 2.0 mm^3^) than at V3 (12.6 ± 2.0 mm^3^) (*P* < 0.001, *P* < 0.001), and even significantly smaller at V4 than at V2 (*P* = 0.009). The thick horizontal line in the graph represents the mean and the two thin lines represent the standard deviation. BCVA, best corrected visual acuity; CMT, central macular thickness; MV, macular volume.

BCVA showed significant improvement at V2 (0.30 ± 0.23) than at V1 (0.39 ± 0.29) and at V4 (0.34 ± 0.26) than at V3 (0.48 ± 0.34) (*P* = 0.002, *P* < 0.001), but no significant difference was observed between V2 and V4 (*P* = 0.257) (Fig. 2a). CMT was significantly thinner at V2 (346.8 ± 90.2 µm) than at V1 (495.5 ± 123.8 µm) and at V4 (322.2 ± 95.7 µm) than at V3 (536.5 ± 166.0 µm) (*P* < 0.001, *P* < 0.001). However, no significant difference was observed between V2 and V4 (*P* = 0.140) (Fig. 2b). MV was significantly smaller at V2 (11.6 ± 2.0 mm^3^) than at V1 (12.6 ± 1.9 mm^3^) and at V4 (11.2 ± 2.0 mm^3^) than at V3 (12.6 ± 2.0 mm^3^) (*P* < 0.001, *P* < 0.001), and even significantly smaller at V4 than at V2 (*P* = 0.009) (Fig. 2c).

Supplemental Table 1 shows a comparison of the best outcomes during the period of conventional anti-VEGF treatment with those at V4. BCVA at V4 was significantly worse than the best BCVA (0.16 ± 0.31) during conventional anti-VEGF treatment (*P* = 0.004). Although there was no significant difference between CMT at V4 and the thinnest CMT (314.7 ± 73.8 µm) during conventional anti-VEGF treatment (*P* = 0.935), CMT at V4 was thinner than the thinnest CMT during the period of traditional anti-VEGF treatment in 12 eyes (52.2%). Although there was no significant difference between MV at V4 and the smallest MV (11.0 ± 1.3 mm^3^) during conventional anti-VEGF treatment (*P* = 0.260), MV at V4 was smaller than the smallest CMT during the period of traditional anti-VEGF treatment in 9 eyes (39.1%).

**Figure 3:**
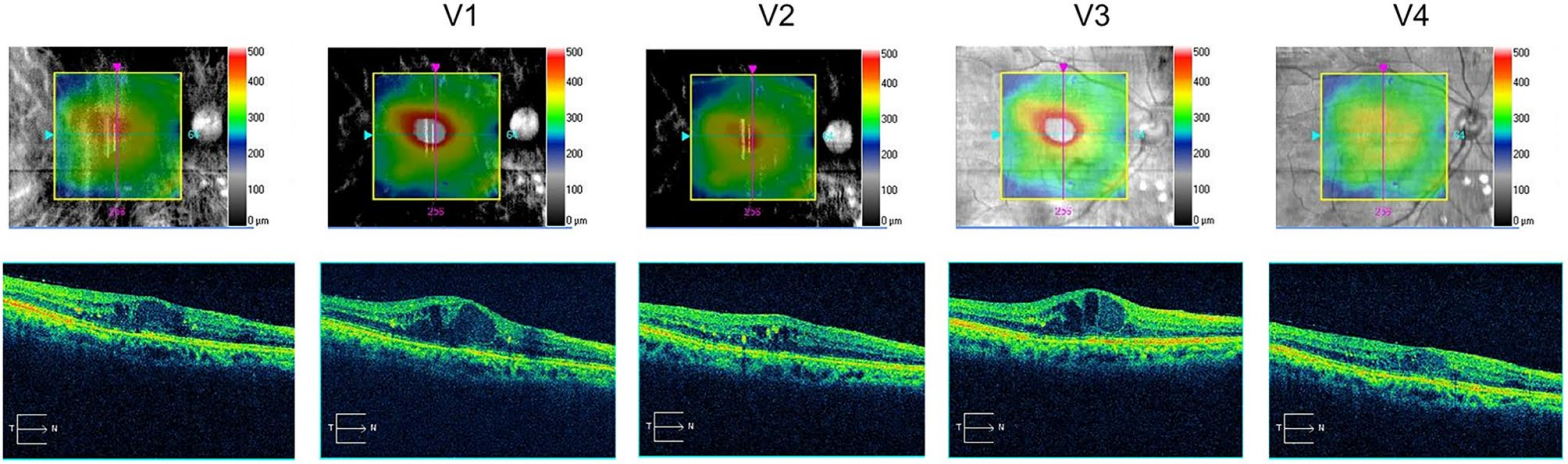
A representative case of switching to IVBr due to inadequate efficacy. The left panel shows the point at which CMT was at its lowest during intravitreal aflibercept treatment; however, CMT was not satisfactory at 396 µm. Before and after the last intravitreal injections of aflibercept before switching to IVBr, CMT decreased only slightly from 515 µm (V1) to 459 µm (V2). CMT before the first IVBr injection was 459 µm (V3), but one month after the first IVBr injection, it significantly reduced to 312 µm (V4). CMT, central macular thickness; IVBr, intravitreal injection of brolucizumab.

The representative case shown in Fig. 3 depicts that CMT was not less than 315 µm during four intravitreal injections of aflibercept, but it thinned to 312 µm at V4, i.e., one month after switching to IVBr.

### Adverse events

**Figure 4:**
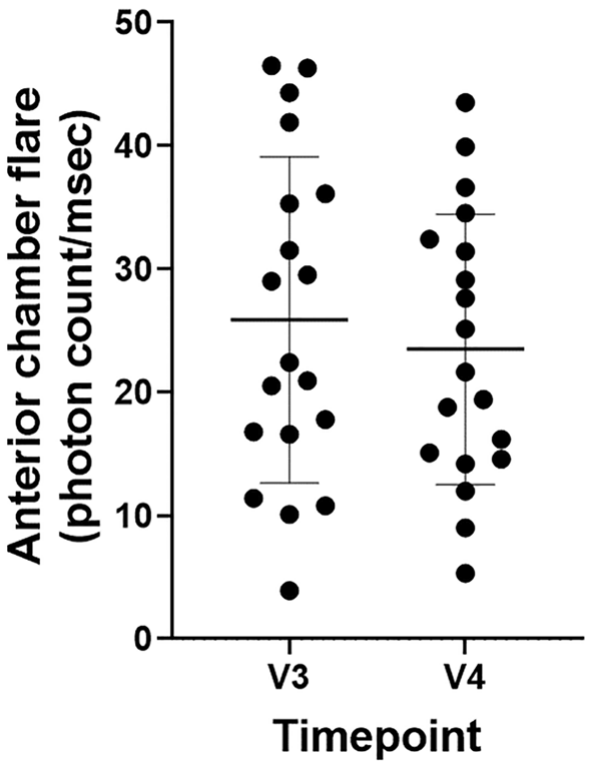
Comparison of anterior chamber flare values between V3 and V4. There was no significant change in the anterior chamber flare values between V3 and V4 (25.6 ± 14.6 vs. 24.0 ± 11.5 photon count/ms; *P* = 0.543) in the 20 eyes for which examination data were available. The thick horizontal line in the graph represents the mean and the two thin lines represent the standard deviation.

None of the patients showed any evidence of IOI. No significant changes in anterior chamber flare values was observed between V3 and V4 (25.6 ± 14.6 vs. 24.0 ± 11.5 photon count/ms; *P* = 0.543) in the 20 eyes for which examination data were available (Fig. 4). No other adverse events were identified during the observation period.

## Discussion

In the present study, we retrospectively evaluated the before and after effects of switching from conventional anti-VEGF therapy to IVBr in a real-world clinical setting. Prior conventional anti-VEGF therapy and IVBr significantly reduced CMT and MV at one month, demonstrating anatomical effects. In addition, MV at one month was found to reduce significantly with IVBr than with anti-VEGF therapy. These results suggest that brolucizumab may reduce retinal fluid more efficiently than anti-VEGF agents such as ranibizumab and aflibercept. We also compared the best morphology during the treatment period with conventional anti-VEGF and the first morphology after switching to IVBr. No statistically significant differences were observed between CMT and MV in terms of morphology. However, 12 eyes (52.2%) in CMT and nine (39.1%) in MV showed the best morphological improvement during the treatment course.

Brolucizumab is a novel VEGF inhibitor with a molecular weight of 26 kDa. It is smaller than the commercially available ranibizumab (48 kDa) and aflibercept (97–115 kDa). Brolucizumab can be concentrated at 120 mg/mL due to its high solubility^19^. Therefore, the binding affinity of brolucizumab for VEGF is higher than that of ranibizumab and aflibercept. This molecular feature may be one of the reasons for favorable morphological improvement with IVBr observed in this study.

Both conventional anti-VEGF therapy and IVBr showed significant improvement in BCVA as well as CMT and MV at one month. However, when the best BCVA during the treatment period was compared with the BCVA with conventional anti-VEGF, the BCVA at one month after IVBr showed poor results, which was statistically significant. Since the participants in the current study had been treated with anti-VEGF for an extended period (mean: 11.6 anti-VEGF injections), many of them likely suffered from irreversible visual impairment, including damage to the external limiting membrane or ellipsoid zone and macular ischemia. To avoid this, switching to a more effective anti-VEGF therapy may be essential before irreversible morphological damage occurs.

The incidence rate of IOI after IVBr in the HAWK and HARRIER phase III trials of brolucizumab for AMD was 4.6%^15^. This was higher than those after ranibizumab and aflibercept (1.5% and 0.5–1.1%, respectively)^19^. Significant increaments in the expression of pro-inflammatory bioactive substances, such as intercellular adhesion molecule (ICAM)-1, interleukin (IL)-6, and monocyte chemotactic protein (MCP)-1, have been reported in the vitreous of patients with DME compared to controls^20^. Since DME is an inflammatory disease, there has been a concern that IOI may be more common in DME than in controls. Therefore, based on this fact, IOI may be more common in DME than in AMD. However, the reported IOI rates were 4.7% and 3.7% for the 3 mg and 6 mg brolucizumab groups, respectively, in KESTREL, and 1.7% in the 6 mg brolucizumab group in KITE, which was not significantly different from those in nAMD^11^. Although there were no cases of IOI or retinal vasculitis, such as increased anterior chamber flares, one month after IVBr in this study, further longitudinal evaluation of the efficacy and safety of brolucizumab is warranted.

This study had a few limitations. The small number of patients included and the study's retrospective design are significant limitations. The reason for switching to IVBr was the expectation of an extended dosing interval, which accounted for six eyes (26.0%). This study only evaluated a short period (1 month after brolucizumab administration); thus, it was not possible to determine whether an extended dosing interval was required. Further research and a large prospective study are needed to validate the present conclusions. In addition, we focused only on anatomic and functional outcomes after brolucizumab administration and did not evaluate specific molecular or immunologic parameters. Therefore, we could not analyze the preceding pathophysiology at the molecular level.

In summary, one month after switching from conventional anti-VEGF to IVBr therapy for DME, both CMT and MV were significantly reduced. After one month of treatment, MV was significantly lower for IVBr than prior anti-VEGF therapy. In addition, the best morphological improvement was achieved in several cases, even though brolucizumab was administrated only once. Our results indicate that brolucizumab may be a viable treatment option for DME patients considering switching from conventional anti-VEGF agents for various reasons, such as poor response or inability to extend dosing intervals. Long-term analyses are required to confirm the efficacy and safety of brolucizumab in routine clinical settings.

## Supplementary Information

Supplementary Table 1.

## Data Availability

The datasets generated during and/or analyzed during the current study are available from the corresponding author upon reasonable request.
