# Supplementary material for: Evaluating initial responses to brolucizumab in patients undergoing conventional anti-VEGF therapy for diabetic macular edema: a retrospective, single-center, observational study
Source: Sci Rep. 2023 Jul 5;13:10901. doi: 10.1038/s41598-023-37726-5 (PMC10322981; doi:10.1038/s41598-023-37726-5)
Supplement: Supplementary file 1 — Supplementary Table 1. [file 41598_2023_37726_MOESM1_ESM.docx]

Supplemental Table 1. Comparison of best outcomes during conventional anti-VEGF treatment with outcomes at V4 and reasons for switching to IVBr

| Case  No. | Best  BCVA during the period of conventional anti-VEGF treatment | BCVA  at V4 | Thinnest CMT during the period of conventional anti-VEGF treatment | CMT at V4 | Smallest  MV during the period of conventional anti-VEGF treatment | MV  at V4 | Reasons for switching to IVBr |
| --- | --- | --- | --- | --- | --- | --- | --- |
| 1 | 0.22 | 0.52 | 316 | 332 | 11.8 | 11.0 | inadequate efficacy |
| 2 | 0.22 | 0.40 | 396 | 312 | 10.8 | 10.4 | inadequate efficacy |
| 3 | 0.00 | 0.05 | 282 | 240 | 11.9 | 11.0 | others |
| 4 | -0.08 | 0.05 | 356 | 559 | 10.5 | 11.5 | inadequate efficacy |
| 5 | 0.22 | 0.40 | 294 | 307 | 12.0 | 11.3 | extended dosing interval |
| 6 | 0.00 | 0.10 | 401 | 342 | 12.1 | 11.3 | inadequate efficacy |
| 7 | 0.00 | 0.40 | 223 | 238 | 11.2 | 10.9 | extended dosing interval |
| 8 | 0.40 | 0.40 | 242 | 248 | 9.0 | 9.0 | others |
| 9 | 0.05 | 0.15 | 401 | 370 | 14.9 | 13.3 | inadequate efficacy |
| 10 | -0.08 | 0.05 | 210 | 298 | 9.5 | 9.7 | others |
| 11 | 0.00 | 0.70 | 338 | 350 | 11.4 | 11.5 | inadequate efficacy |
| 12 | -0.08 | 0.30 | 303 | 298 | 11.2 | 11.4 | others |
| 13 | 0.10 | 0.15 | 261 | 252 | 10.2 | 10.4 | others |
| 14 | 0.15 | 0.82 | 211 | 223 | 9.5 | 9.5 | others |
| 15 | -0.08 | 0.10 | 260 | 252 | 10.3 | 10.7 | extended dosing interval |
| 16 | 0.30 | 0.15 | 402 | 311 | 10.5 | 10.3 | inadequate efficacy |
| 17 | 0.00 | 0.10 | 356 | 337 | 12.3 | 12.1 | inadequate efficacy |
| 18 | 0.70 | 0.70 | 445 | 388 | 12.2 | 12.0 | inadequate efficacy |
| 19 | 1.00 | 0.52 | 393 | 527 | 9.3 | 17.0 | others |
| 20 | 0.82 | 0.70 | 397 | 506 | 11.0 | 15.4 | others |
| 21 | -0.18 | 0.40 | 279 | 235 | 9.2 | 8.8 | extended dosing interval |
| 22 | -0.08 | 0.70 | 244 | 224 | 10.1 | 9.5 | extended dosing interval |
| 23 | 0.10 | 0.05 | 228 | 262 | 11.7 | 8.9 | extended dosing interval |
| Average | 0.16 | 0.34 | 314.7 | 322.2 | 11.0 | 11.2 | - |
| SD | 0.31 | 0.26 | 73.8 | 95.7 | 1.3 | 2.0 | - |

The measurements that were better compared to the best results with conventional anti-VEGF treatment are shown in bold.

BCVA, best corrected visual acuity; CMT, central macular thickness; IVBr, intravitreal injection of brolucizumab; MV, macular volume; SD, standard deviation; VEGF, vascular endothelial growth factor
